# Using Large Language Models to Automate Data Extraction From Surgical Pathology Reports: Retrospective Cohort Study

**DOI:** 10.2196/64544

**Published:** 2025-04-07

**Authors:** Denise Lee, Akhil Vaid, Kartikeya M Menon, Robert Freeman, David S Matteson, Michael L Marin, Girish N Nadkarni

**Affiliations:** 1 Department of Surgery, Icahn School of Medicine at Mount Sinai, 10 Union Square East, Suite 2L, New York, NY, 10003, United States, 1 212 241 2891; 2 Charles Bronfman Institute for Personalized Medicine, Icahn School of Medicine at Mount Sinai, New York, NY, United States; 3 Department of Statistics and Data Science, Cornell University, Ithaca, NY, United States

**Keywords:** natural language processing, large language model, artificial intelligence, thyroid cancer, endocrine surgery, framework, privacy, medical, surgical pathology, report, NLP, medical question

## Abstract

**Background:**

Popularized by ChatGPT, large language models (LLMs) are poised to transform the scalability of clinical natural language processing (NLP) downstream tasks such as medical question answering (MQA) and automated data extraction from clinical narrative reports. However, the use of LLMs in the health care setting is limited by cost, computing power, and patient privacy concerns. Specifically, as interest in LLM-based clinical applications grows, regulatory safeguards must be established to avoid exposure of patient data through the public domain. The use of open-source LLMs deployed behind institutional firewalls may ensure the protection of private patient data. In this study, we evaluated the extraction performance of a locally deployed LLM for automated MQA from surgical pathology reports.

**Objective:**

We compared the performance of human reviewers and a locally deployed LLM tasked with extracting key histologic and staging information from surgical pathology reports.

**Methods:**

A total of 84 thyroid cancer surgical pathology reports were assessed by two independent reviewers and the open-source FastChat-T5 3B-parameter LLM using institutional computing resources. Longer text reports were split into 1200-character-long segments, followed by conversion to embeddings. Three segments with the highest similarity scores were integrated to create the final context for the LLM. The context was then made part of the question it was directed to answer. Twelve medical questions for staging and thyroid cancer recurrence risk data extraction were formulated and answered for each report. The time to respond and concordance of answers were evaluated. The concordance rate for each pairwise comparison (human-LLM and human-human) was calculated as the total number of concordant answers divided by the total number of answers for each of the 12 questions. The average concordance rate and associated error of all questions were tabulated for each pairwise comparison and evaluated with two-sided *t* tests.

**Results:**

Out of a total of 1008 questions answered, reviewers 1 and 2 had an average (SD) concordance rate of responses of 99% (1%; 999/1008 responses). The LLM was concordant with reviewers 1 and 2 at an overall average (SD) rate of 89% (7%; 896/1008 responses) and 89% (7.2%; 903/1008 responses). The overall time to review and answer questions for all reports was 170.7, 115, and 19.56 minutes for Reviewers 1, 2, and the LLM, respectively.

**Conclusions:**

The locally deployed LLM can be used for MQA with considerable time-saving and acceptable accuracy in responses. Prompt engineering and fine-tuning may further augment automated data extraction from clinical narratives for the provision of real-time, essential clinical insights.

## Introduction

Surgical pathology reports contain narrative data essential for hospital-based comprehensive cancer surveillance databases and the real-time understanding of patient staging, recurrence risk, clinical trial eligibility, and personalized treatment options. Large-scale national databases such as the Surveillance, Epidemiology, and End Results (SEER) program and the National Cancer Database (NCDB) include population-level histologic data necessary to study rare cancers and underrepresented patient subpopulations [1,2]. However, the large-scale extraction of key surgical oncologic insights contained within unstructured free pathology text is limited by the need for labor-intensive, manual review by human reviewers [3]. The maintenance of comprehensive cancer databases often requires skilled registrars who are trained to synthesize and extract information from patient narrative reports according to established data collection standards [4]. However, constantly updated guidelines, time-intensive labor for data extraction, and inevitable human error are challenges to coordinate cancer data collection.

Advances in natural language processing (NLP) techniques have sought to address challenges in the efficiency and accuracy of data abstraction. Efforts have included applying NLP methods to extract pain scores in patients with cancer undergoing radiation [5], classify metastatic phenotypes from radiology reports of patients with colorectal cancer [6], and identify recurrence status in patients with hepatocellular carcinoma [7]. Traditional NLP methods, however, require specific domain expertise and may be error-prone based on statistical or rule-based approaches [8]. Large language models (LLMs) power a new generation of natural language processing (NLP) whereby deep neural networks are trained on a massive corpus of human text that are then deconstructed into vectorized embeddings that depict linguistic relationships in a numerical format appropriate for easy analysis [9]. Popularized by ChatGPT and its user-friendly question-and-answer interface, LLMs are poised to transform the scalability of clinical NLP downstream tasks such as medical question answering (MQA) and data mining. LLMs may also enhance the ability to rapidly and accurately extract key information from surgical pathology reports [10].

However, as new LLM-based clinical applications are being increasingly explored, there is a substantial risk of protected health information (PHI) breaches without regulatory safeguards in place [11]. Ethical, privacy, and regulatory constraints preclude the transfer of PHI across the public domain through widely used proprietary LLM services (eg, ChatGPT, Gemini, and Claude) that can generate automated responses for MQA. However, significant health care resources (ie, utilization fees and institutional agreements) would be required to ensure the protection of PHI while accessing the largest state-of-the-art models such as GPT4 via a private cloud service provider (eg, Microsoft Azure OpenAI). Such considerable upfront costs and service agreements may preclude hospital systems without financial and IT resources from utilizing the largest and most state-of-the-art LLMs.

**Figure 1.**
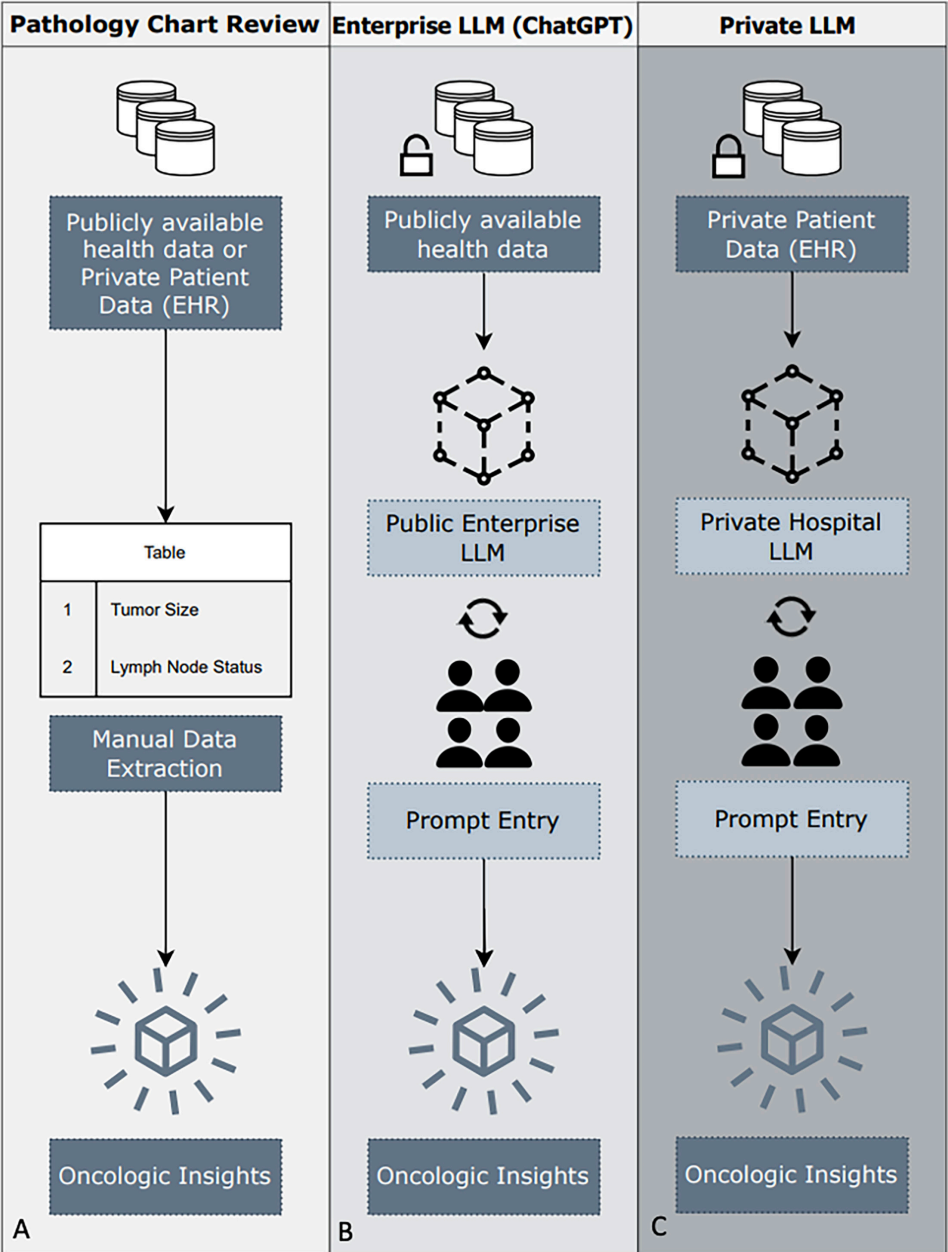
Overview of medical data extraction workflows: (A) Pathology chart review: The traditional approach of manual data extraction from publicly available databases or private electronic health records (EHRs) to obtain predetermined oncologic insights. (B) Enterprise large language model (LLM, such as ChatGPT): Due to regulatory constraints, only publicly available data may be shared with enterprise LLMs. Prompt entry and question curation are used to gain oncologic insights. (C) Private LLMs: EHR data can be shared with a local hospital LLM, and prompt entry with question curation can be used to gain oncologic insights. EHR: electronic health record.

The rapid development in LLM technology has led to the development of many open-source models (eg, Llama 3.1 and Google Gemini) both comparable in performance to GPT4 and deployable on an institution’s existing computing clusters. The use of a smaller, locally deployed LLM may help ensure that protected health information is maintained and democratize access to LLM technologies at medical centers where larger, private LLMs are inaccessible. In this study, we investigated the use of a locally deployed, open-source LLM to extract key staging and recurrence risk information from thyroid surgical pathology reports. We compared its performance to the gold standard of medically trained human reviewers. Although we utilize this for a single-use case, our work serves as a practical pathway to enable individual medical centers to utilize current LLM technology for clinical NLP tasks in a privacy-protecting manner (Figure 1).

## Methods

### Ethical Considerations

This study was approved by the Institutional Review Board of the Icahn School of Medicine at Mount Sinai (no. 22‐00347). Informed consent was waived given the retrospective nature of the study. To ensure confidentiality of patient data, only authorized research study personnel were permitted access to patient data. Study identification numbers were assigned to patients included in the study for deidentification.

### Study Population

**Figure 2.**
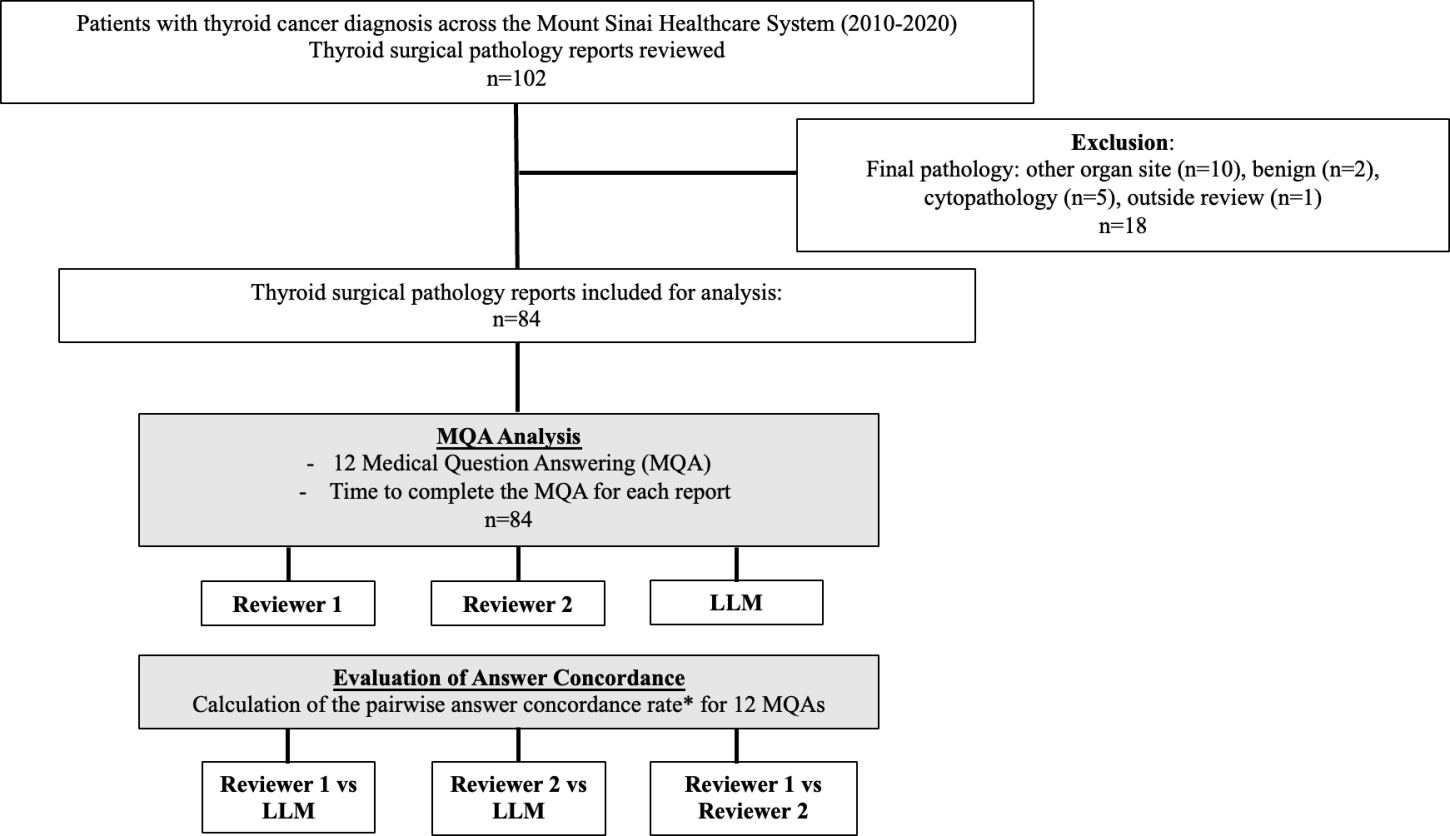
Flowchart of the study design and analysis. *The concordance rate was calculated as the total number of concordant answers/total number of answers for each of the 12 medical question answering (MQA).

We queried the Mount Sinai Data Warehouse for a cohort of adult patients (>18 years old) with *ICD-9* (International Classification of Diseases, 9th revision) and *ICD-10* (International Classification of Diseases, 10th revision) diagnosis codes for thyroid cancer and who underwent thyroid surgery between 2010 and 2022. We reviewed 102 surgical pathology reports from 102 patients and excluded reports if they were from other organ sites (n=10), benign (n=2), cytopathology (n=5), or outside review (n=1). We included 84 reports for analysis. The study flowchart is shown in Figure 2.

### Large Language Model

We used the publicly available, open-source, and parameter-efficient FastChat-T5 3B-parameter LLM for our analysis. FastChat-T5 is a smaller model that requires significantly less computing, making it accessible in resource-constrained environments. This characteristic was essential for our study, as we wanted our findings to be easily replicable across institutions with varying levels of computational resources. In many real-world scenarios, particularly in health care or academic settings, access to high-end hardware or expensive cloud services is limited, and the need for locally deployable solutions behind firewalls is critical due to privacy concerns. FastChat-T5 provides a balance between performance and resource efficiency, making it a practical choice in these contexts. This LLM was deployed on existing hospital servers, which avoided the utilization of expensive cloud services and external transfer of patient data. The ready availability of such software has increased the use of open-source LLMs. The practice of deploying smaller models on local hardware aligns with the latest trends from leading LLM providers that are exploring the deployment of models on mobile devices.

In addition, our goal was to evaluate how a smaller, open-source model performs in comparison to human reviewers, who are generally considered the gold standard in tasks such as document review and content evaluation. We selected FastChat-T5 to explore whether such a parameter-efficient model could offer comparable performance to humans, without requiring highly specialized code or expensive infrastructure for running more recent and resource-intensive models, such as those in the LLaMA (large language Meta AI) family.

A limitation all LLMs have is the amount of context they may process at once, and for FastChat-T5, this limit is 2048 tokens. For reports of lengths greater than what the model could accommodate, we split the report text into 1200-character-long segments, followed by converting each of these segments into machine-readable numerical representations called embeddings. As embeddings encode meaning, calculating similarity scores using the cosine similarity metric between segment embeddings and posed questions allowed us to retrieve the pieces of text most directly related to the content of the question. As such, three segments with the highest similarity scores were integrated to create the final context for the LLM. This context was made part of a plain language question for the LLM, alongside a question it was directed to answer.

We selected the top three segments with the highest similarity scores for several key reasons. First, using multiple segments allows us to enrich the context provided to the LLM, ensuring that it has access to a broader and more diverse set of relevant information. By incorporating three segments, we increase the likelihood that important details from different parts of the document are included, without overwhelming the model’s context window.

Additionally, focusing on the top three ensures that we strike a balance between the relevance and the diversity of information. Using just one segment might omit crucial context, while using too many could dilute the focus on the most pertinent details. The number three was chosen empirically, as it allowed for a good compromise between feeding the LLM with enough useful content and avoiding the risk of both exceeding usable context and introducing unrelated or redundant information that could confound the model’s operation. We did experiment with other segmentation strategies, including combining two or four closest segments, but the performance gains were inferior to those of the three-segment approach. In our preliminary trials, while combining more distinct segments could introduce variety, this approach led to a slight reduction in overall accuracy due to the introduction of less relevant or tangential information.

Finally, embedding models with cosine similarity offers an efficient and robust way to evaluate how closely each segment relates to the posed question, ensuring that the selected segments are not just textually similar but semantically aligned with the user’s query. This method enhances the precision of the context provided to the LLM, resulting in more accurate and relevant answers. The source code can be found at [12] under a GPLv3 license.

The prompt template is available in Multimedia Appendix 1.

### Development of MQA and the Evaluation of Concordance

We formulated 12 questions with expert clinical input that extracted key information for the assessment of the thyroid cancer staging and recurrence risk on the AJCC-TNM staging system (American Joint Committee on Cancer Tumor-Node-Metastasis, 8th edition) according to the American Thyroid Association Recurrence Risk Stratification System [13,14]. Two study authors (DL and KMM) reviewed 84 thyroid surgical pathology reports and recorded answers to each of the 12 questions and the time taken to complete the answers for each report. Then, we used the LLM to answer the same questions. For every question, we determined if the answers were concordant between Reviewer 1 and the LLM, between Reviewer 2 and the LLM, and between the two reviewers. The concordance rate for each pairwise comparison (human-LLM and human-human) was then calculated as the total number of concordant answers divided by the total number of answers for each of the 12 questions (Figure 2). The average concordance rate and associated error (calculated here as the SD) for all questions were tabulated for each pairwise comparison and evaluated with two-sided *t* tests.

## Results

We report the sample LLM responses and the concordance rates between reviewers and the LLM for each question in Table 1. A total of 1008 questions were answered for the 84 thyroid surgical pathology reports. Reviewers 1 and 2 were concordant at an average (SD) overall rate of 99% (1%; 999 out of 1008 questions) with disagreement on 9 answers. Reviewers 1 and 2 took an average (SD) of 2 (0.6) minutes and 1.4 (0.4) minutes to respond to each pathology report and a total of 170.7 minutes and 115 minutes for all reports, respectively. The LLM was concordant with reviewers 1 and 2 at an average (SD) overall rate of 89% (7%; 896 out of 1008 questions) and 89 (7.2%; 903 out of 1008 questions), respectively. The LLM was significantly less accurate than both reviewer 1 (*P*<.001) and reviewer 2 (*P*<.001), but performed at the same level between both reviewers (*P*=.82). The average time to review each report for the LLM was 13.97 seconds/report and 19.56 minutes for all reports. The questions with the highest overall rates of concordant responses were questions requiring binary or categorical data extraction (eg, “Is lymphatic invasion present?”, 100%; “Is vascular invasion present?”, 98.81%; and “Where is the primary cancer located?”, 98.1%). The question with the lowest overall concordance was, “Were cervical lymph nodes present?” at 75%.

**Table 1.** Twelve medical questions and sample large language model (LLM) answers with the rates of concordance between the reviewers and the LLM.

Medical question	Sample LLM answer	Concordance (%)			
		R1,^a^ LLM	R2,^b^ LLM	R1, R2	All 3
Staging tumor (T)					
What is the size of the primary tumor?	The size of the primary tumor was 5.5 cm.	82	84	98	82
Does the tumor extend beyond the capsule of the thyroid?	No, the tumor does not extend beyond the capsule of the thyroid.	90	93	97	90
Where is the primary cancer located (right thyroid lobe, left thyroid lobe, or isthmus)?	The primary cancer is located in the right thyroid lobe.	99	100	99	99
Node (N)					
Were any cervical lymph nodes present?	Yes, cervical lymph nodes were present.	76	100	99	75
If cervical lymph nodes are present, how many lymph nodes were positive for malignancy?	Three lymph nodes were positive for malignancy.	88	88	100	88
Stage (TNM)^c^					
What is the final pathology TNM stage of the thyroid cancer?	The final pathology TNM stage of the thyroid cancer is pT3a.	85	85	100	85
Recurrence risk factor					
Is vascular invasion present?	Yes, vascular invasion is present.	99	99	100	99
Is lymphatic invasion present?	No, lymphatic invasion is not present.	100	100	100	100
What was the histology of the primary cancer?	The histology of the primary thyroid cancer was papillary carcinoma.	87	87	100	87
Was there a variant of thyroid cancer such as tall cell, hobnail variant, or columnar cell variant present, indicative of a more aggressive histology?	No, a variant of thyroid cancer such as tall cell, hobnail variant, or columnar cell variant was not present indicative of a more aggressive histology.	89	92	97	89
Was there a second thyroid cancer present?	No, there was no second thyroid cancer present.	82	83	99	82
If there was a second thyroid cancer present, what was the histology?	The histology of the second thyroid cancer was classical variant papillary thyroid carcinoma.	88	88	99	88
Overall questions, % (SD)		89 (7)	89 (7)	99 (1)	89 (7.2)

a R1: Reviewer 1.

b R2: Reviewer 2.

c TNM: Tumor-Node-Metastasis.

## Discussion

We demonstrated and evaluated the extraction performance of a locally deployed, open-source LLM for a specific clinical NLP task. To our knowledge, this is the first study to compare an LLM’s ability to extract key thyroid cancer recurrence and staging information accurately and efficiently from surgical pathology reports using a conversational MQA format. The LLM took 19.56 minutes to evaluate and respond to all pathology reports, whereas it required an additional 187 minutes and 105 minutes for the reviewers to complete the same task—demonstrating a considerable reduction in time. Regarding the accuracy of the responses, we found that the rates of response concordance were higher among questions tasked with simpler binary or categorical responses. The increase in task complexity requiring textual interpretation and inconsistent word prompting, such as asking whether there was “cervical” lymph nodes present, resulted in the lowest rate of concordance. Furthermore, the question regarding the size of the primary tumor also seemed to be relatively straightforward but had an overall concordance rate of only 82%.

The augmentation of poorer-performing MQA may lie in the improvement of prompt engineering—an emerging subfield where domain-specific knowledge and linguistics are optimized to design questions that yield the best-performing response to a task. In addition, more expressive embeddings may help localize relevant text [15]. For example, “cervical” does not appear in most pathological reports verbatim, possibly limiting the model’s ability to respond appropriately to the question regarding the presence of cervical lymph nodes. Moreover, the LLM often incorrectly identified the size of the “primary tumor” and would instead provide a dimension from another specimen in the report, such as the overall thyroid lobe. This response accuracy may also be improved by modifying the question prompt and will be the focus of future work.

Overall, the use of LLMs may be an advancement from early NLP methods that faced limitations such as restrictive data preprocessing and the inability to handle multiple positive diagnoses [16-18]. Studies exploring the ability of LLMs to handle real clinical data have been rapidly growing. Examples include the investigation of the use of LLMs for multiple clinical NLP tasks such as the summarization of magnetic resonance imaging and radiology reports of the knee [19], the simplification of anatomic pathology reports for patient interpretation [20], and data extraction from breast cancer pathology and radiology reports [21]. However, the protection of private patient information must be prioritized with the use of any emerging technology. Safeguards should be established to ensure data breaches can be avoided with the use of LLMs for clinical tasks. The approach of deploying smaller, open-source LLMs behind a health care institution’s own computing resources ensures that researchers across all centers may use this emerging technology while maintaining patient privacy. Additionally, the increased language capacity of the latest generation of LLMs allows institutions to deploy their own data for in-context learning only while potentially achieving a reasonable performance.

The limitations of this study include the use of a single institutional dataset without comparison with an external dataset to evaluate the generalizability of the LLM performance. Differences in language, reporting style, and description length of surgical pathology reports of different cancer types may also affect the ability of a smaller LLM such as the open-source FastChat-T5 used in this study to accurately extract the requested data using prompts. Additionally, while the intention of the study was to compare the performance of a smaller, open-source LLM to the gold standard of human reviewers, a comparison of its performance with larger, private LLMs such as GPT should be explored in future studies, after data have been confirmed to be entirely deidentified.

This study demonstrated that a locally deployed, open-source LLM is capable of automating data extraction faster than human reviewers who need to perform a manual review of surgical pathology reports. However, although the responses between the LLM and expert reviewers reached concordance rates of 88‐89%, the degree of error by the LLM precludes its exclusive use to populate cancer statistics databases. Until LLMs demonstrate comparable performance to humans, the use of LLMs for clinical NLP tasks still requires considerable oversight by human reviewers. As a human-assistance tool, the role of LLMs may lie in improving the time efficiency of populating clinical databases. As methods of prompt engineering and fine-tuning improve, we envision that LLMs will eventually allow medical institutions to harness cutting-edge advances in NLP for timely and privacy-preserving MQA data extraction from pathology reports and other clinical narratives for the provision of real-time, essential oncologic insights.

## Supplementary material

10.2196/64544 Multimedia Appendix 1 Transcript of prompts.

## Abbreviations

ICD: International Classification of Diseases
LLaMA: large language Meta AI
LLM: large language model
MQA: medical question answering
NCDB: National Cancer Database
NLP: natural language processing
PHI: protected health information
SEER: Surveillance, Epidemiology, and End Results
