# Supplementary material for: Using Large Language Models to Automate Data Extraction From Surgical Pathology Reports: Retrospective Cohort Study
Source: JMIR Form Res. 2025 Apr 7;9:e64544. doi: 10.2196/64544 (PMC11996145; doi:10.2196/64544)
Supplement: Multimedia Appendix 1 [file formative-v9-e64544-s001.docx]

Supplementary Material:

Prompt Template: with context & questions below extract between “<” and “>”

Use the following pieces of context to answer the question at the end. If you don't know the answer, just say that you don't know, don't try to make up an answer.

<Pieces of context as extracted from the report>

Question: <Question relevant to the text, as asked by clinician>

| Questions Relevant to the Text: Inserted Programmatically to the Prompt Template Above   1. Where is the primary cancer located (right thyroid lobe, left thyroid lobe or isthmus)? |
| --- |
| 1. What was the histology of the primary thyroid cancer? |
| 1. Was there a variant of thyroid cancer such as tall cell, hobnail variant, or columnar cell variant   present indicating more aggressive histology? |
| 1. Was there a second thyroid cancer present? If yes, what was its histology? |
| 1. What was the size of the primary tumor? |
| 1. Were any cervical lymph nodes present? |
| 1. If cervical lymph nodes are present, how many lymph nodes were positive for malignancy? |
| 1. Is vascular invasion present? |
| 1. Is lymphatic invasion present? |
| 1. Does tumor extend beyond the capsule of the thyroid (ie, is extrathyroidal extension present)? |
| 1. What is the final pathology TNM stage of the thyroid cancer? |
